# SFCW Radar with an Integrated Static Target Echo Cancellation System

**DOI:** 10.3390/s21175829

**Published:** 2021-08-30

**Authors:** Danijel Šipoš, Dušan Gleich

**Affiliations:** Faculty of Electrical Engineering and Computer Science, University of Maribor, Koroska Cesta 46, 2000 Maribor, Slovenia; dusan.gleich@um.si

**Keywords:** SFCW radar, ground-penetrating radar, through-wall imaging radar, clutter cancellation, echo cancellation, clutter suppression, echo suppression

## Abstract

Continuous Wave (CW) radars systems, especially air-coupled Ground-Penetrating Radar (GPR) or Through-Wall Imaging Radar (TWIR) systems, echo signals reflected from a stationary target with high energy, which may cause receiver saturation. Another effect caused by reflection of stationary targets is noticeable as background within a radargram. Nowadays, radar systems use automatic gain control to prevent receiver saturation. This paper proposes a method to remove stationary targets automatically from the received signal. The method was designed for a radar system with a moving platform, with an assumption that the distance between the surface and target is constant. The design is proposed of an SFCW radar with an integrated system for real-time multiple static target Echo Cancellation (EC). The proposed EC system removes the static target using active Integrated Circuit (IC) components, which generate the corresponding EC signal for each frequency step of the SFCW radar and sum it with the received echo signal. This has the main advantage of removing even multiple echoes at any distance, and excludes the need for a high-dynamic-range receiver. Additionally, the proposed system has minimal impact on the radar size and power consumption. Besides static target removal, the antenna coupling can be removed if the signal appears to be constant. The operating frequency was selected between 500 MHz and 2.5 GHz, due to the limitation of the used electronic components. The experimental results show that the simulated target’s echo using a cable with a known length could be suppressed to up to 38 dB. Experimental results using a moving radar platform and the real environment scenario with static and dynamic targets, show that the proposed EC system could achieve up to 20 dB attenuation of the static target. The system does not affect any other target of interest, which can even move at any distance during the measurement. Therefore, this could be a promising method for further compact implementation into SFCW radars, or any other radar type that generates CW single frequencies.

## 1. Introduction

A radar is a nondestructive sensor that was first used for military applications. Nowadays, radars can be found in industrial applications, healthcare and medical applications, remote sensing, and subsurface investigation [1]. Depending on how the radar signal is generated, radars can be divided into time and frequency domain groups [2]. First mentioned was the impulse radar, which works in the time domain [3], and its advantage is its simple hardware design and signal processing. The representatives of the frequency domain radars are the Frequency-Modulated Continuous Wave (FMCW) radar [4] and the Stepped-Frequency Continuous Wave (SFCW) radar [5]. The popularity of these radars has increased, as they demodulate a high-frequency signal to an Intermediate Frequency (IF) signal, which does not require high-speed Analog-to-Digital Converters (ADCs). Additionally, these radars have higher sensitivity and a wider dynamic range compared with time domain radars [6,7]. Therefore, their use can been found in different fields, and the current focus is highly on automotive applications [8,9,10].

As both the SFCW and FMCW radars work in the same domain, the acquired data bins have to be transformed from the frequency to the time domain. Nevertheless, despite the data acquisition similarity, the signal generator and receiver work differently. Therefore, the SFCW radar shows better performance in applications where a lower operating frequency band has to be used, and, at the same time, high resolution is required with a low noise figure. A drawback compared with FMCW radars is the acquisition time, which is longer. A popular use of the SFCW radar principle is in Ground-Penetrating Radar (GPR) applications [11,12,13,14,15], where small objects‘ detection was presented. Due to the fact that the signal travels through a highly lossy medium—soil—the sensitivity of such a sensor is crucial. Another scenario where the observed scene and required performance are similar would be in through-wall imaging radar applications [16,17,18,19]. The sensor sensitivity also has to be high, since the signal is attenuated strongly through the wall, as it may contain metal pieces or multiple air slots. An even more drastic scenario would be in an air-coupled antenna system [20,21,22,23], where the signal has to travel through an additional medium—air. This kind of system has become especially popular, since no physical contact is needed between the investigated medium and the antenna. In this scenario, not only does attenuation cause a problem, but also the fact that the Radar Cross-Section (RCS) of the observed surface can be several times larger than the RCS of the target of interest; RCS is a measure of the object‘s ability to reflect the signal back to the direction of the receiver and does not relate directly on the object‘s area, but also considers frequency, aspect angle, and polarization [24]. This explains the large differences in the single-echo amplitudes. By increasing transmit power to amplify a weak echo signal, this can cause the receiver saturation because of the strong echo signals. To find a solution for this problem, large RCS targets—e.g., ground surface reflection or reflections from the wall—have to be categorized. A main assumption is that the ground surface or the wall is homogeneous. In this case, even if the radar moves, the distance to the surface could be controlled easily, and remain relatively constant during the whole measurement. Due to this, such a target could be categorized as a static target. Other targets of interest that appear during the measurement are then considered as dynamic, which separates them from static targets.

In this paper, we present a complete SFCW radar design with an integrated Echo Cancellation (EC) system, which is able to cancel multiple static targets with a combination of active and passive components. The first implementation of digital CW signal cancellation was presented and implemented in 1994 [25], where a bandwidth of 200 MHz could be achieved. Recent work has shown that the bandwidth could already be increased to above a few GHz [26] with the use of measuring equipment such as Vector Network Analyzers (VNAs). The proposed system is based on the previously developed compact SFCW radar [27], and its extension represents an integrated EC system that affects the size of the radar and the power consumption minimally. The proposed method is highly suitable for battery-powered devices and applications where weight is critical.

The EC signal is generated with an RF synthesizer [28] that is synchronized with the transmitter and can perform a phase shift all the way up to 2π radian or more, without modifying the hardware itself. As the amplitude of the echo signal can change at each frequency step, the EC system must match it in order to achieve the highest efficiency. Therefore, a digital attenuator is used to perform attenuation of the EC signal in uniform steps. Before the EC system can be used, a calibration procedure is needed and requires a measurement of the static scene. This step provides the information about the required EC signal phase and amplitude through the whole used frequency spectrum.

The proposed EC method was applied to the SFCW radar using implementation on multiple boards to speed up the prototyping process. The performance of the EC system was tested through a frequency band between 500 MHz and 2.5 GHz, which covers a popular range for GPR or through-wall imaging applications. The first experiment was performed with direct connection of the transmitter and receiver using a cable with a known length. This allowed us to measure the system delay and simulate a single target. Further experiments have been performed in a scene with a large homogeneous static target and a smaller dynamic target. TEM flared antennas were used for transmitting and receiving the signal. To obtain a B-scan, these were moved over a scene with the use of a motorized rail.

The proposed system is not only suitable for an SFCW radar system, but also for any other CW system, e.g., Doppler radar. An important fact to note is that the system cannot be implemented in another pre-existing SFCW radar, since the transmitter and EC system depend on each other. The bandwidth could be increased further, but was limited due to the components, such as RF amplifiers and antennas.

## 2. SFCW Radar

The principle of SFCW radar is based on stepping through a definite frequency span in uniform frequency steps Δf [5]. The range resolution performance is equal to the very similar and widely used FMCW radar, and also relates to the bandwidth *B* as

(1) $$\Delta R=\frac{v_{m}}{2B}$$

where $v_{m}=1/\sqrt{\varepsilon_{m}\mu_{m}}$ is the velocity of the Electromagnetic (EM) wave through a given medium, and its permittivity $\varepsilon_{m}$ and permeability $\mu_{m}$ define the value.

The output frequency of such a radar is defined as

(2) $$f_{n}=f_{0}+n\Delta f\;n=0,1,2,\cdots ,N$$

where $f_{0}$ is the start frequency and *N* defines the number of frequency steps. The value *N* also defines the theoretical maximum range which can be obtained as

(3) $$R_{max}=\Delta RN$$

At least, *N* defines the measure time of one complete frequency sweep, or also named range bin ($t_{bin}$) as

(4) $$t_{bin}=Nt_{f}$$

where $t_{f}$ is the time of a single-frequency transmission. In practice, this parameter is the SFCW radar‘s biggest disadvantage, since to detect long-range targets, the number *N* needs to be high and $t_{f}$ cannot be short as the hardware needs a certain amount of time to lock the desired frequency.

If a target is present, the receiver will obtain an echo signal with the time delay τ, corresponding with the two-way travel time to the target, and an amplitude $A_{n}$. The signal in a complex-valued form can be expressed as

(5) $$s_{n}\left(\omega_{n},t\right)=A_{n}\exp\left(-j\left(\omega_{n}\left(t-\tau \right)\right)\right)$$

where $\omega_{n}=2\pi f_{n}$. The signal could be sampled directly with a high-speed ADC. To avoid this, the signal can be demodulated further to an In-Phase (I) and Quadrature (Q) signal, using a quadrature-demodulator, where the output can be described in a vector form as

(6) $$C_{n}\left(\omega_{n},\tau \right)=I_{n}\left(\omega_{n},\tau \right)+jQ_{n}\left(\omega_{n},\tau \right)=\exp\left(-j\phi_{n}\right)$$

$\phi_{n}$ represents the phase between the transmitted and received signals and is related directly with the time delay as $\tau =\phi_{n}/\omega_{n}$. To represent the target as a single response, an Inverse Discrete Fourier Transform (IDFT) has to be applied on the vector *C*, where the result can be expressed as

(7) $$y_{n}=\frac{1}{N}\sum_{i=0}^{N-1}C_{i}\exp\left[\frac{j2\pi ni}{N}\right]$$

Since the value *N* defines the number of range points, the length of vector *C* can be increased by adding zeros. This will result in an increase of the range points and provide better range accuracy. The result of the IDFT needs to be represented in the absolute form as $|y_{n}|$. A synthetic pulse will occur in the location corresponding with the time delay caused by the target and make it visible.

### Echo Response

**Figure 1:**
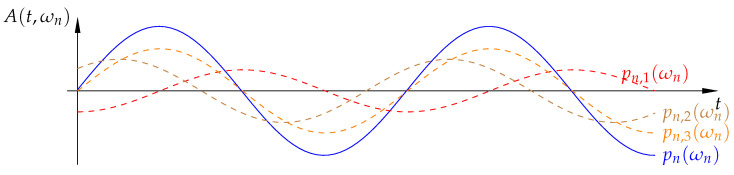
Multiple single echo signals from three targets $p_{n,1}\left(\omega_{n}\right)$, $p_{n,2}\left(\omega_{n}\right)$, $p_{n,3}\left(\omega_{n}\right)$ and the corresponding echo sum $p_{n}\left(\omega_{n}\right)$.

The received radar’s EM signal is time delayed for the two-way travel time to the target. Equation (5) describes the received signal for a single target for the SFCW radar principle. In practice, the received signal is a sum of multiple targets, which is described with Equation (8).

(8) $$p_{n}\left(\omega_{n},t\right)=\sum_{l=1}^{U}A_{n,l}\exp\left(-j\left(\omega_{n}\left(t-\tau_{l}\right)\right)\right)=F_{n}\exp\left(-j\left(\omega_{n}\left(t-\tau_{tot}\right)\right)\right)$$

where *U* defines the number of all targets, $F_{n}$ is the total amplitude, and $\tau_{tot}$ is the total time delay of the summed echo signals. Figure 1 shows an example of multiple echo signals and their sum from targets at different locations for frequency $f_{n}$. To take the most advantage of the receiver dynamic range, the received signal has to be scaled through the whole available ADC range. This can be achieved by accordingly amplifying or attenuating the transmit or receive signals. Otherwise, if the received single echo signals with the smallest and highest amplitudes have a great difference between each other, the sensitivity of such a system is greatly limited.

## 3. Echo Cancellation

In the case of a time-domain radar, an echo that is strong enough to saturate the receiver does not have a critical effect on the final radargram compared with the frequency-domain radar. An impulse radar is a representative of time-domain radars, and transmits short pulses that are time-delayed by a relation between the EM speed in a given medium and the distance at the receiver’s site [29]. Different target echoes, therefore, do not interfere with each other at the receiver, even if, at a certain point, some of them would saturate the receiver. This is not the case in Continuous Wave (CW) radars such as the SFCW or FMCW radars. The transmitting time is much longer than the echo response time of the most distant target, which means that all echoes are summed together and cannot be separated once received by the antenna.

At a certain frequency $\omega_{n}$, it is impossible to separate a single echo from the summed signal $p_{n}\left(\omega_{n},t\right)$. One of the possible solutions would be to subtract—cancel it physically, directly on the receiver input. This could be achieved with physical summing $p_{n}\left(\omega_{n},t\right)$ with a replica version, which we name the Echo Cancellation (EC) signal, is matched by amplitude, and has a phase difference of π radian. For a simplified scenario where only stationary targets are present, the summed result is described with Equation (9).

(9) $$\begin{array}{ccc} r_{n}\left(\omega_{n},t\right) & = & p_{n}+o_{n}\\ & = & F_{n}\exp\left(-j\left(\omega_{n}\left(t-\tau_{tot}\right)\right)\right)\\ & + & G_{n}\exp\left(-j\left(\omega_{n}\left(t-\tau_{tot}\right)+\pi +\theta_{n}\right)\right) \end{array}$$

where $G_{n}$ is the EC signal amplitude, which is expected to be closest to $G_{n}=F_{n}$, and $\theta_{n}$ is the phase offset between the EC signal and transmit signal. If there is no difference in the distances between the transmitted signal and the EC signal, then $\theta_{n}=0$ radian. In an ideal case scenario, the result would be $r_{n}\left(\omega_{n},t\right)=0$.

In a real environment where the radar platform is usually moving, it would be challenging to track and cancel only specific echoes, as the signal is changing constantly with the distance. In applications such as GPR or through-wall imaging, the most problematic echoes are stationary. Considering this, an echo signal of this type of target would remain the same through the whole measurement, even if the radar platform is moving. The EC System, therefore, removes only static echoes and keeps dynamic targets visible. The proposed EC system is not only limited to GPR or through-wall imaging applications, but can also be used for similar problem solving. For instance, instead of reducing antenna coupling with an RF circulator [30], the proposed system achieves a similar effect, as the coupling has the same performance as an echo signal of a static target.

In practice, the applications that use the EC system should be calibrated each time the static scene changes, e.g., the distance of the radar has changed. Calibration is the process where a static scene is recorded, and the appropriate phase and echo power are obtained for each frequency. If, during the measurement, any other captured dynamic echo appears, after the system calibration, it will change the value $p_{n}\left(\omega_{n},t\right)$, and make the target visible.

## 4. The Proposed Hardware Implementation and System Workflow

**Figure 2:**
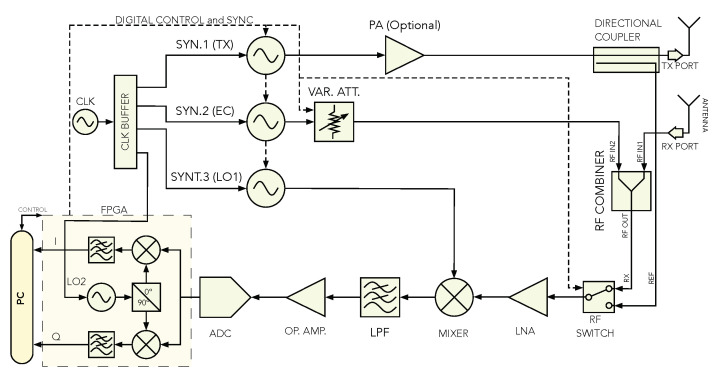
Block diagram of the proposed super-heterodyne architecture SFCW radar with integrated EC system.

**Figure 3:**
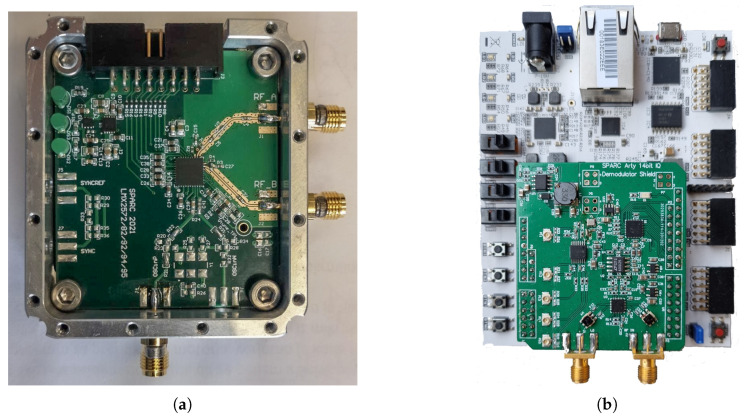
Main developed hardware of the proposed super-heterodyne architecture SFCW with EC system (**a**) The synthesizer board (**b**) The digital IQ demodulator board with the FPGA processing unit.

The EC system can be implemented as an extension to the previously developed SFCW radar [27], with a receiver’s design changes. The proposed system’s block diagram is shown in Figure 2. The main part of the radar systems are the three frequency synthesizers boards (SYN. 1—TX synthesizer; SYN. 2—EC synthesizer; SYN. 3—LO1 synthesizer), which generate all the necessary signals. The hardware for SYN 1-3 is the same, therefore, the single board is shown in Figure 3a. All frequency synthesizer boards use the same clock buffer as a reference source, which enables phase synchronization, and performs a phase shift of the signal within a few picoseconds. To achieve phase synchronization, an additional line has to be provided to deliver a time-critical phase sync pulse. Figure 2 shows the TX synthesizer that generates the transmit signal, the EC synthesizer that generates the EC signal, and the LO1 synthesizer that is used on the receiver and generates the Local Oscillator (LO1) signal source for the Intermediate Frequency (IF) down-converter. The transmitted signal is filtered by a Low-Pass Filter (LPF) to suppress higher harmonics. In the proposed system, the transmitted signal is, after filtering, coupled directly with the antenna, but could, if needed, also be amplified optionally with a Power Amplifier (PA).

The EC signal has, compared with the received signal (echo signal), a higher amplitude, and needs to be attenuated. A digital attenuator was used to achieve variable attenuation. Control of all digital devices such as the synthesizers, the ADC, the attenuator, and the RF switch is performed using a Field-Programmable Gate Array (FPGA).

The receiver is designed in a super-heterodyne architecture [31] with an analog IF down-converter and a digital Intermediate-Quadrature (IQ) demodulator, where Figure 3b shows the receiver and processing unit. Before the signal is coupled with the Low-Noise Amplifier (LNA), a digital switch selects the acquisition of the RX port or REF port. The REF port is connected directly with the TX port over a coaxial cable, while the RX port guides the sum between the echo signal received with the antenna and EC signal. Either the signal that goes through the RX port or the REF port is then amplified with an LNA. The RX—REF signal is demodulated further to an IF signal with frequency $f_{if}=2$ MHz, and filtered additionally with an LPF to reject the higher harmonics that are caused by the transmitter. Afterwards, the IF signal is amplified and sampled with a 14-bit at 40 MSa/s ADC. The selected sampling rate matches the value of the frequency, which also feeds all three synthesizers and the FPGA. As all devices share the same clock, coherence is ensured, which is necessary for the IQ demodulation [27]. Further, IQ demodulation and low-pass filtering are performed on an FPGA. The much higher ADC sample rate compared with the frequency $f_{if}$ additionally prevents aliasing, which could potentially be caused by the higher harmonics of the synthesizers. The final result is a complex-valued vector $s_{iq,n}$, where the number of frequency steps defines its length.

### 4.1. The Proposed Echo Cancellation and Calibration Workflow

A calibration procedure is proposed to achieve the best performance of the EC system. The calibration procedure is the most important step that has to be accomplished before starting the normal operation mode. The efficiency of the EC can be determined directly through the magnitude $|s_{acq}|$ of the acquired signal $s_{acq}$. The goal of the EC algorithm at this stage is to remove the transmitter’s signal at the receiver’s site. This is achieved by amplitude matching and phase estimation between the signals generated with the EC synthesizer and TX synthesizer at the point where they are coupled with the combiner. In this paper, we propose using a brute force method to determine the unknown parameters $\theta_{n}^{\prime }=\theta_{n}+\pi$, which represent the phase shift offset of the EC synthesizer with an additional phase shift of π radian, $a_{n}$, which represents the attenuation amount of the digital attenuator in dB. Attenuation of the EC signal is performed, as we assume that the amplitude of the EC synthesizer is always greater than the amplitude of the TX synthesizer. The phase shift step Δθ and attenuation step Δa are software selected, and limited by the hardware capability. The calibration operation mode is described in Algorithm 1.
**Algorithm 1:** Echo Cancellation calibration1:Set the RF switch port to RX input2:**for**$f_{n}=f_{0}+n\Delta f$; n=0,1,…,N−1**do**3:    Set TX synthesizer and EC synthesizer to frequency $f_{n}$4:    Set LO1 synthesizer to frequency $f_{n}+f_{if}$5:    Set predefined value of digital attenuator6:    **for** Set EC synthesizer phase shift to ${\hat{\theta }}_{m}=m\Delta \theta$; m=0,1,…,P−1 **do**7:        Store data to a temporary array $b_{m}=\left|s_{acq}\right|$8:    **end for**9:    Find phase shift index at minimum magnitude as
$$d=arg min\{b_{0},\ldots ,b_{P-1}\}$$10:    Change EC synthesizer phase shift to value ${\hat{\theta }}_{d}$ and store it as $\theta_{n}^{\prime }={\hat{\theta }}_{d}$11:    **for** Set digital att. to ${\hat{a}}_{x}=a_{off}-x\Delta a$; x=0,1,…,K−1 **do**12:        Store data to a temporary array $b_{x}=\left|s_{acq}\right|$13:    **end for**14:    Find attenuation shift index at minimum magnitude as
$$z=arg min\{b_{0},\ldots ,b_{K-1}\}$$15:    store the attenuator value as $a_{n}={\hat{a}}_{z}$16:**end for**

After a complete frequency sweep, when $\theta_{n}^{\prime }$ and $a_{n}$ are obtained, the EC system can be enabled in normal operation mode.

### 4.2. The Proposed Normal Operation Mode Workflow

In normal operation mode, the proposed radar sweeps through the available frequency band and acquires complex-valued data. The EC system can be disabled or enabled, where, in the latter case, the calibration values $\theta_{n}^{\prime }$ and $a_{n}$ are used. In normal operation mode, an additional step is required, and it is not related with the EC system. The reference signal is needed because the signals of the LO1 synthesizer and TX synthesizer have different frequencies and phase lock times. Therefore, for each frequency step, the initial phase of the generated signal is unknown. The problem was solved by using a coaxial cable to feed the REF port of the RF switch, and guide the signal to its common output port, since the cable has a fixed length and causes a constant delay between the TX and RX ports. The obtained complex-valued signal represents the reference signal $s_{ref}$. The normal operation mode of this radar can be described with Algorithm 2.

The ${(}^{*})$ denotes the complex conjugate operator—it should be noted that the result $s_{iq,n}$ does not correlate directly with the actual distance to the target, but has a constant offset caused by the reference line length and the phase response of the directional coupler. However, since it is only an offset, it does not have any other influence on the quality of the measurement. Due to this, in the experimental results, the radargram range is also expressed as relative.

### 4.3. Hardware Overview

The SFCW radar with EC was developed using widely available IC components, which can be controlled digitally. For speeding up the prototyping process, the radar was divided into the following sub-boards:

- Transmitter (TX) synthesizer—Synthesizer board 1;
- Echo Cancellation (EC) synthesizer—Synthesizer board 2;
- Local Oscillator 1 (LO1) synthesizer—Synthesizer board 3;
- Receiver board (IF down-converter, IQ demodulator, ADC, clock buffer);
- FPGA board;
- Digital attenuator board.

**Algorithm 2:** Normal operation mode1:**for**$f_{n}=f_{0}+n\Delta f$; n=0,1,…,N−1**do**2:    Set TX synthesizer and EC synthesizer to frequency $f_{n}$3:    Set LO1 synthesizer to frequency $f_{n}+f_{if}$4:    **if** EC is enabled **then**5:        Change EC synthesizer phase shift to $\theta_{n}^{\prime }$6:        Change the digital attenuator value to $a_{n}$7:    **else**8:        Disable the EC synthesizer9:    **end if**10:    Set the RF switch port to REF input11:    Obtain $s_{ref}=s_{acq}$12:    Set the RF switch port to RX input13:    Obtain $s_{rx}=s_{acq}$14:    Calculate the complex-valued IQ signal as $s_{iq,n}=s_{rx}^{*}/s_{ref}^{*}$15:**end for**

All boards that include high-frequency RF signals were realized on a 4-Layer substrate for simpler impedance matching between wave-guide ports. Additional small modules were used, such as an RF Combiner, RF switch, directional coupler, amplifier, and attenuator. The hardware was designed according to the block scheme shown in Figure 2. A Transverse Electromagnetic (TEM) flared antenna [32], was used to transmit and receive the RF signal. A summary of the selected relevant parameters of the SFCW radar with an integrated EC system is shown in Table 1.

As stated before, the start frequency $f_{start}$ and stop frequency $f_{stop}$ were limited mainly by the amplifiers and antennas. With a different selection of these components, the frequencies could be extended to the range between 12.5 MHz to 6.4 GHz. The IF frequency has a constant value of $f_{if}$ = 2 MHz and is generated indirectly with LO1, whose output is defined as $f_{lo1,n}=f_{n}+f_{if}$.

The PC was connected to the FPGA board, and communicates over a serial communication. This communication includes the control of digital configurable devices, such as transferring the raw IQ data back to the PC. The acquired data are then processed further on the PC. The PC itself could be replaced by using a storage device, where the needed configuration would be read-out directly by the FPGA, and also the acquired data would be written back to it.

## 5. Experimental Results

The experimental results provide a proof of concept of the proposed EC system with a simulated target and a static reflection. The Experimental Results section is divided into two parts. The first part of the measurements shows the efficiency of the proposed EC system without antennas. Instead of using antennas, the signal is coupled directly over a coaxial cable. Additionally, the experimental setup includes TX and RX antennas, where a real environment configuration is set up. This includes a homogeneous static target and a target of interest.

### 5.1. Calibration and System Delay Estimation with a Direct Cable Coupling

**Figure 4:**
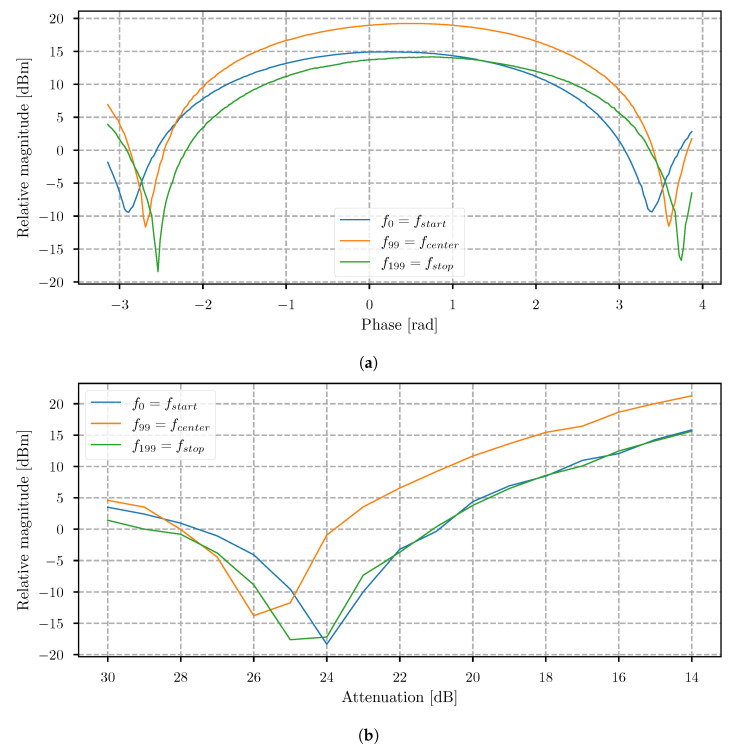
(**a**) Obtained magnitude $|s_{iq,n}|$ of the summed EC and RX signal for frequencies $f_{start}$, $f_{center}$, and $f_{stop}$, when (**a**) a phase shift was performed and (**b**) an attenuation shift was performed.

This stage provides the information on how efficiently the signal can be canceled or suppressed for each frequency step when the procedure of Algorithm 1 is followed. A coaxial cable of 1 m-length was used to simulate the delayed echo signal without influencing the frequency characteristic as the antenna would. The first step was to estimate the phase shift of the EC signal by changing the phase in steps of 0.025 radian for a total value of 7 radian, and the total phase shift exceeded one period (2π), which would be the minimal needed shift. The magnitude minimum for each frequency step was determined after storing all the acquired data. For comparison of the EC success rate at different frequencies for the first stage, referred to as phase shifting, Figure 4a shows the obtained result for $f_{start}$, $f_{center}$, and $f_{stop}$, where $f_{center}=f_{N/2}$.

**Figure 5:**
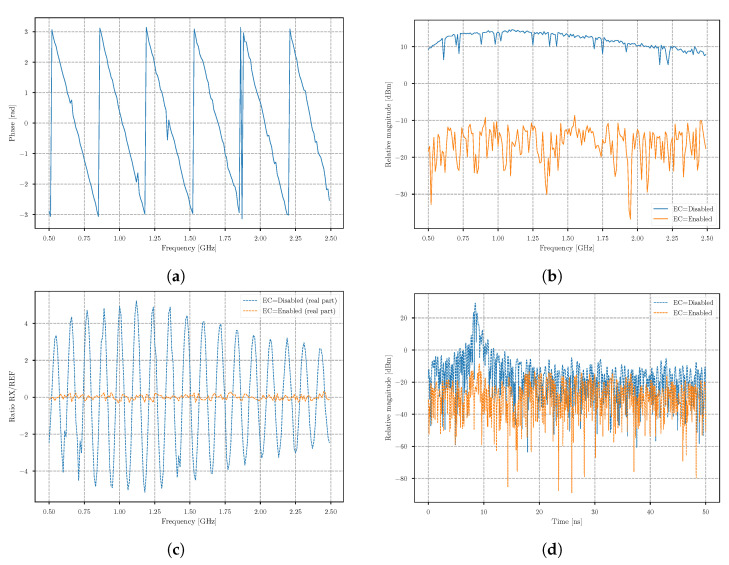
Obtained results when the TX synthesizer and the EC synthesizer are connected with a 1 m-long coaxial cable (**a**) Phase shift through the frequency. (**b**) Magnitude measured with the receiver when EC is disabled and enabled. (**c**) The real part of the measured complex-valued signal with the receiver when EC is disabled and enabled. (**d**) The signal magnitude in the time domain of the acquisition with the receiver when EC is disabled and enabled.

If we then vectorize the location of the minimum at each frequency, we obtain the phase between the TX synthesizer and EC synthesizer at the point when the signals are coupled with the RF combiner and can be defined as $\theta_{tx/ec}$. The result is shown in Figure 5a. The obtained measurement is also required later for faster calibration (see Section 5.2). The second stage of the calibration procedure is attenuation stepping, and can be performed once the phase $\theta_{n}^{\prime }$ for each frequency step is estimated. To prevent damaging the receiver, the attenuation start value was set to the maximum of 31 dB, and then decreased in steps of 1 dB for 17 iterations. Figure 4b shows the magnitude response when the attenuation was decreased by frequencies $f_{start}$, $f_{center}$, and $f_{stop}$. The results showed that, for each frequency step, the result was a quasi-convex-like function. The optimal match was found to be at the magnitude minimum, meaning that the compensation of amplitudes was also determined. Furthermore, this value already provides the information of the EC system efficiency at frequency $f_{n}$. The experimental result, presented in Figure 5b, shows the acquired frequency sweep with EC disabled and enabled. The experimental results showed that the cancellation rate was between 19 dB and 25 dB. This value is the most important parameter of the EC system, as it indicates the amount of power that could be increased on the transmitter without saturating the receiver. Furthermore, Figure 5c shows the same result, where the real part of the IQ data is shown, and in Figure 5d, the corresponding transform of the IQ data to the time domain is shown. The result in the time domain indicates a spike, which occurs at the value related to the signal traveling time of the simulated echo. If we compare the results between an enabled and disabled EC system, we can observe that the difference in magnitudes is about 45 db. The system not only allows for using higher transmit power, but also improves the contrast, where no further software background subtraction would be needed.

### 5.2. Phase Offset Estimation

**Figure 6:**
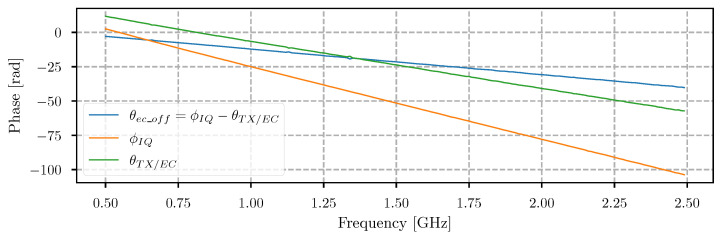
Unwrapped phase offset of the EC synthesizer path $\phi_{ec\_off}$ estimated from $\phi_{iq}$ and $\theta_{tx/ec}$.

The procedure, explained in Section 5.1, where the phase is estimated for each frequency step, is time consuming, especially if the phase shift step is small. The time can be reduced if we have already estimated the needed phase offset for a static scene when the radar is in normal mode. The calibration procedure for estimation of the time delay between the TX synthesizer and EC synthesizer at the point of combining was presented in Section 5.1. This means that this time delay can also be expressed with a phase defined as $\theta_{tx/ec}$. The proposed method suggests to obtain a normal range bin $s_{iq,n}$ with the EC system disabled, and using the exact setup as it was used in the calibration procedure. The phase between the transmitted and received signals was introduced with Equation (6), and is now calculated as $\phi_{iq,n}=\arg\left(s_{iq,n}\right)$. This phase provides the information about the time delay caused by the 1 m-long cable, which connects the TX and RX ports. The time delay of the EC synthesizer line at the point of the combiner input is determined if the phase is subtracted $\theta_{ec\_off,n}=\phi_{iq,n}-\theta_{tx/ec,n}$. Once the estimated parameters are provided, we could estimate any static scene by calculating the sum of the phase and the phase delay of the EC signal line as $\theta_{n}^{\prime }=\phi_{iq,n}+\theta_{ec\_off,n}$. The result of the proposed implementation is shown in Figure 6, where all phases were unwrapped. This method provides the ability to reduce the calibration time, since only a single sweep has to be captured for the phase shift. The digital attenuator value calculation could be based on a similar approach and additionally reduce calibration time, but was unreliable with testing, and, therefore, needs the same procedure as was already presented in Algorithm 1. Future experiments will be based on the proposed simplified calibration method.

### 5.3. Echo Cancellation in a Real Environment

The EC system performance cannot be validated using only a simulated static target, as was presented using the coaxial cable. This is due to the fact that the coaxial cable does not affect the signal over frequency noticeably, as the antenna would. In a scenario when an antenna would be used, this means that the RX port of the RF combiner is fed with the signal received from the antenna, and the EC port is fed directly over a coaxial cable from the EC synthesizer. Since the frequency response between a cable and an antenna is different, we expect that the EC efficiency will decrease. To confirm this and evaluate that the EC system is still suited for use with antennas, a real-environment experiment has been carried out, where a large surface static target and a target of interest were used. The experiment was performed using a moving radar platform, as this tests the performance under more severe conditions compared with a static platform.

**Figure 7:**
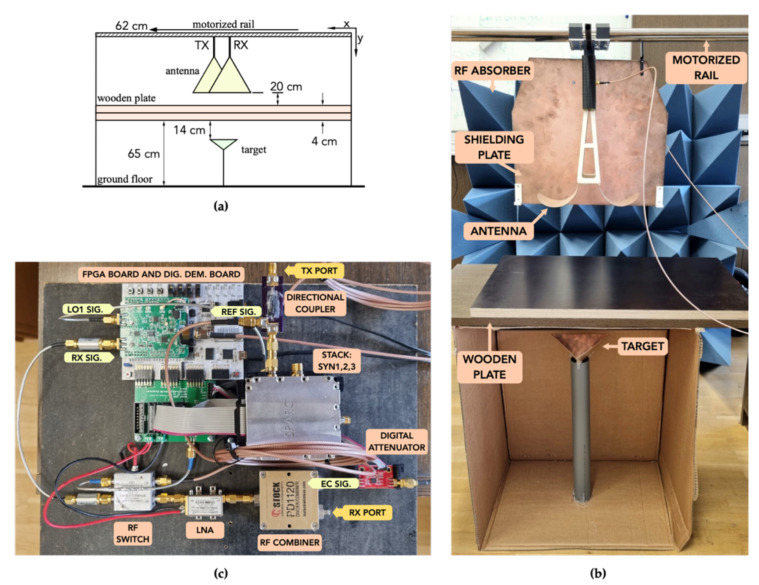
(**a**) Detailed data of the experimental setup. (**b**) Experimental setup. (**c**) Connection setup of the complete SFCW radar with EC system.

**Figure 8:**
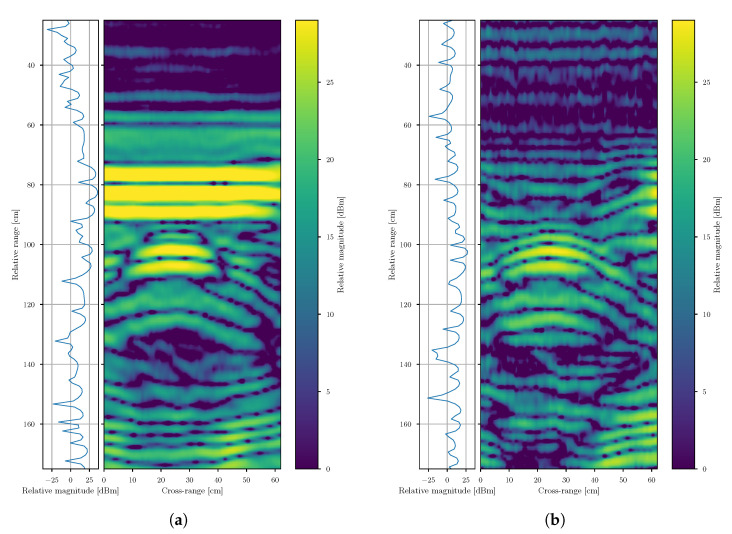
(**a**) Radargram with a homogeneous static target and a target of interest with EC system disabled and the corresponding slice left at 24 cm Cross-range. (**b**) Radargram with a homogeneous static target and a target of interest with EC system enabled and the corresponding slice left at 24 cm Cross-range.

Two TEM-flared antennas were used in the presented experiment. The antennas were separated by a fixed distance and mounted on a motorized rail. Two wooden plates were placed below the antennas in parallel with the motorized rail. The total thickness of the wooden plate was 4 cm, which served as the static target. A metal corner reflector was positioned below the wooden plates, which represented a target of interest. The cross-range distance step was chosen to be 1 cm, and the total cross-range was 62 cm, as shown in Figure 7a. The experimental setup is shown in Figure 7b and the assembled SFCW radar with EC system in Figure 7c. An external PC had simultaneous control of the motorized rail and radar system to obtain a B-Scan measurement in normal operation mode, following Algorithm 2. Figure 8a shows the radargram when the EC system was first disabled. From the result, we can observe that the strongest static echo appears at the relative range of about 82 cm, and was present as expected through the whole cross-section. Following are two more static echoes, before and after the strongest. This could occur because of some small air-gap between the wooden plates, or they are side-lobes of the strongest peak. At a cross-range of about 58 cm, we see that the magnitude of the static signal starts decreasing. The reason lies in the fact of too-short wooden plates. At the relative range of about 100 cm, we can observe the echo response of the target of interest, which, as also expected, has a parabolic shape. The range is expressed as relative, because the offset includes the antenna cable length and the path difference between the TX–RX port and reference port. Figure 8b shows the measurement performed using the same parameters and setup, with the difference that the EC system was enabled. The system was previously calibrated at a cross-range of 10 cm to ensure the whole antenna radiation pattern covered the static target completely. From the results, we can observe that the static targets were removed with high success. From a cross-range of about 55 cm, it appears as though EC effectiveness would decrease. The reason is that, at this point in the previous measurement with the EC disabled, the magnitude decreased, which means that, at this point, the EC system will start generating an artificial target. Therefore, it is important that the static target is present through the whole scan. Similar to the previous measurement, we can observe that the target of interest remains at the same location and is clearly visible. The target visibility has even increased, not by magnitude, but the sense that the parabolic shape has extended.

**Figure 9:**
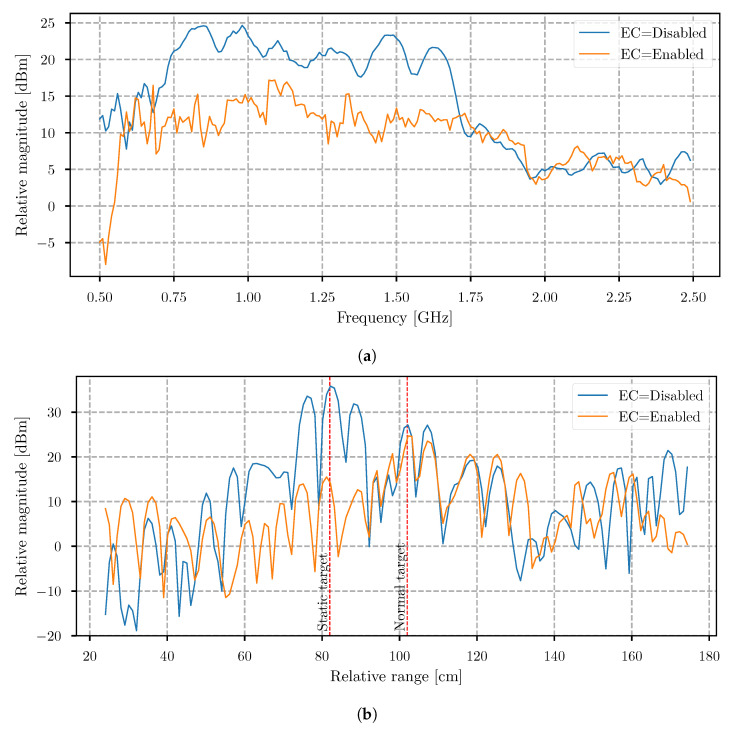
(**a**) Average magnitude of the data in the frequency domain to the cross-range 55 cm, when EC is disabled and enabled. (**b**) Average magnitude of the data in the time domain to the cross-range 55 cm, when EC is disabled and enabled.

To have a better oversight on the comparison when the EC system was enabled and disabled, data averaging in the cross-range direction was performed to obtain only a single dimension result (see Figure 9a). Instead of showing just a single measurement bin, an average reduces the noise, and offers better insight of the EC system efficiency. In the results, we noticed that, when the EC system was disabled, the magnitude dropped significantly at frequencies lower then 750 MHz and higher than 1.75 GHz, and was most likely caused by the antenna frequency characteristic. In this region, when the EC system is enabled, the efficiency of the EC lowers, and, at some points, even adds gain. However, in the other part of the frequency spectrum where the magnitude was higher, the EC efficiency was the same, and lowered the magnitude by about −12 dB.

The radargram shows that the frequency components at which the signals were not suppressed successfully do not have a visible impact on the final result because the static target has obviously been removed, as shown in Figure 9b. However, in this average, the data captured from a further 55 cm of cross-range are excluded, as the wooden board was no longer present. From our point of interest, most relevant is only the distance where the static target was present, and it is visible that the magnitude was about 20-dB lower.

## 6. Conclusions

The SFCW radar design with an integrated EC system is proposed in this paper. The system is especially suitable for GPR and through-wall imaging applications, where the difference in the echo power of different targets is usually large. The methodology of operation of such a system is known and has been presented in recent works, but the main difference is reflected in the implementation of such a system. This means that the system could mostly be suitable for power and size critical applications. Nowadays, this is crucial, due to the need to implement various sensors into, e.g., robots or Unmanned Aerial Vehicles (UAVs).

The experimental results showed that the proposed method is able to suppress the stationary signals using the proposed procedures. To verify the operation of the system in a real environment, a measurement was performed that included the use of antennas and a polygon with a static target. As an antenna with a certain frequency response was used, the results were not expected to be as successful as with the coaxial cable. However, the measurements showed that the removal of reflections was still successful at frequencies where the magnitude was high, which confirms the efficiency of the proposed system in practice. Attention must be given to a scenario where the stationary target disappears or the RCS changes. In these cases, the EC system would operate in the opposite manner and add artificial targets.

Future work could also include techniques that are currently implemented in automotive radars, such at the orthogonal noise waveforms method, and, additionally, increasing the visibility of targets with small RCS [33]. Furthermore, adding an antenna frequency response block would increase the success of the EC system and allow use of a higher transmit power.

Since the system used CW signals, this means that the proposed method could also be applied to other radars that use the same working principle.

## Figures and Tables

**Table 1:** Summary of the main SFCW radar with EC hardware parameters.

Parameter	Value
Start frequency ($f_{start}$)	500 MHz
Stop frequency ($f_{stop}$)	2.5 GHz
Frequency step (Δf)	10 MHz
Intermediate Frequency (IF)	2 MHz
IF sampling frequency	14-bit at 40 MSa/s
Transmitter power	−10 dBm
RF receiver gain	30 dB
IF gain	12 dB
Power consumption	5 W

## Data Availability

Not applicable.
